# Supplementary material for: Study of the Genetic Variants in BRCA1/2 and Non-BRCA Genes in a Population-Based Cohort of 2155 Breast/Ovary Cancer Patients, Including 443 Triple-Negative Breast Cancer Patients, in Argentina
Source: Cancers (Basel). 2021 May 31;13(11):2711. doi: 10.3390/cancers13112711 (PMC8198763; doi:10.3390/cancers13112711)
Supplement: Supplementary file 1 [file cancers-13-02711-s001.zip › cancers-1233266-supplementary.pdf]

cancers

MDPI

Article

Study of the Genetic Variants in *BRCA1/2* and Non-*BRCA* Genes in a Population-Based Cohort of 2155 Breast/Ovary Cancer Patients, Including 443 Triple-Negative Breast Cancer Patients, in Argentina

Angela R. Solano, Pablo G. Mele, Fernanda S. Jalil, Natalia C. Liria, Ernesto J. Podesta and Leandro G. Gutierrez

Table S1. Pathogenic variants detected in the women probands of the cohort, n = 181 for patients and n = 18 for healthy carriers (TNBC probands are in the main part of this publication).

ID	Study	Age	Tumor	Gene	Exon / Intron	HGVS c.	HGVS p.
AN100	AsP	47	NO	*BRCA1*	2	c.68_69del	Glu23Valfs*17
AN101	AsP	49	NO	*BRCA1*	2	c.68_69del	Glu23Valfs*17
AN102	*BRCA1/2*	39	NO	*BRCA1*	5	c.181T>G	Cys61Gly
AN103	*BRCA1/2*	41	NO	*BRCA1*	11	c.1088del	Asn363Ilefs*11
AN104	*BRCA1/2*	32	NO	*BRCA1*	11	c.1360_1361del	Ser454*
AN105	*BRCA1/2*	43	NO	*BRCA1*	11	c.1687C>T	Gln563*
AN106	*BRCA1/2*	54	NO	*BRCA1*	11	c.2255T>G	Leu752*
AN107	*BRCA1/2*	61	NO	*BRCA2*	11	c.2808_2811del	Ala938Profs*21
AN108	*BRCA1/2*	25	NO	*BRCA1*	11	c.3770_3771del	Glu1257Glyfs*9
AN109	*BRCA1/2*	23	NO	*BRCA1*	11	c.3800T>G	Leu1267*
AN110	*BRCA1/2*	31	NO	*BRCA1*	18	c.5091_5092del	Cys1697*
AN111	*BRCA1/2*	49	NO	*BRCA1*	19	c.5177_5180del	Arg1726Lysfs*3
AN112	MLPA	33	NO	*BRCA1*	19i-20i	c.(5193+1_5194-1)_(5277+1_5278-1)del	

AN113	*BRCA1/2*	54	NO	*BRCA2*	11	c.5351dup	Asn1784Lysfs*3
AN114	AsP	43	NO	*BRCA2*	11	c.5946del	Ser1982Argfs*22
AN115	AsP	45	NO	*BRCA2*	11	c.5946del	Ser1982Argfs*22
AN116	AsP	40	NO	*BRCA2*	11	c.5946del	Ser1982Argfs*22
AN117	*BRCA1/2*	60	NO	*BRCA2*	24	c.9382C>T	Arg3128*
AN118	AsP	50	BrCa	*BRCA1*	2	c.68_69del	Glu23Valfs*17
AN119	AsP	66	BrCa	*BRCA1*	2	c.68_69del	Glu23Valfs*17
AN120	AsP	41	BrCa	*BRCA1*	2	c.68_69del	Glu23Valfs*17
AN121	AsP	45	BrCa	*BRCA1*	2	c.68_69del	Glu23Valfs*17
AN122	AsP	69	BrCa	*BRCA1*	2	c.68_69del	Glu23Valfs*17
AN123	AsP	41	BrCa	*BRCA1*	2	c.68_69del	Glu23Valfs*17
AN124	AsP	77	OvCa	*BRCA1*	2	c.68_69del	Glu23Valfs*17
AN125	*BRCA1/2*	45	BrCa	*BRCA1*	2	c.68_69del	Glu23Valfs*17
AN126	*BRCA1/2*	44	BrCa	*BRCA1*	2	c.68_69del	Glu23Valfs*17
AN127	*BRCA1/2*	40	BrCa	*BRCA1*	2	c.68_69del	Glu23Valfs*17
AN128	*BRCA1/2*	47	BrCa	*BRCA1*	3	c.124del	Ile42Tyrfs*8
AN129	*BRCA1/2*	45	OvCa	*BRCA1*	3i	c.134+1G>C	
AN131	*BRCA1/2*	68	OvCa	*BRCA1*	5	c.181T>G	Cys61Gly
AN132	*BRCA1/2*	35	BrCa	*BRCA1*	5	c.190T>C	Cys64Arg
AN133	*BRCA1/2*	59	OvCa	*BRCA1*	5	c.190T>C	Cys64Arg
AN134	*BRCA1/2*	45	BrCa	*BRCA1*	5	c.211A>G	Arg71Gly

AN135	*BRCA1/2*	43	BrCa	*BRCA1*	5	c.211A>G	Arg71Gly
AN136	*BRCA1/2*	41	BrCa	*BRCA1*	5	c.211A>G	Arg71Gly
AN137	*BRCA1/2*	41	OvCa	*BRCA1*	5	c.211A>G	Arg71Gly
AN138	*BRCA1/2*	33	OvCa	*BRCA1*	5	c.211A>G	Arg71Gly
AN130	*BRCA1/2*	51	OvCa	*BRCA1*	5	c.211A>G	Arg71Gly
AN139	*BRCA1/2*	56	OvCa	*BRCA1*	6	c.250G>T	Glu84*
AN140	*BRCA1/2*	49	OvCa	*BRCA1*	8i	c.547+2T>A	
AN141	*BRCA1/2*	50	OvCa	*BRCA1*	11	c.835delC	His279Metfs*19
AN142	*BRCA1/2*	59	OvCa	*BRCA1*	11	c.876_879del	Thr293Lysfs*4
AN143	*BRCA1/2*	49	BrCa	*BRCA1*	11	c.1067del	Gln356Argfs*18
AN144	*BRCA1/2*	45	BOC	*BRCA1*	11	c.1360_1361del	Ser454*
AN145	*BRCA1/2*	42	OvCa	*BRCA1*	11	c.1360_1361del	Ser454*
AN146	*BRCA1/2*	71	BrCa	*BRCA1*	11	c.1480C>T	Gln494*
AN147	*BRCA1/2*	70	OvCa	*BRCA1*	11	c.1504_1507del	Leu502Serfs*29
AN148	*BRCA1/2*	40	BrCa	*BRCA1*	11	c.1687C>T	Gln563*
AN149	*BRCA1/2*	65	OvCa	*BRCA1*	11	c.1729_1730del	Glu577Ilefs*8
AN150	*BRCA1/2*	55	OvCa	*BRCA1*	11	c.1817del	Pro606Leufs*6
AN151	*BRCA1/2*	55	OvCa	*BRCA1*	11	c.1953dup	Lys652Glufs*21
AN152	*BRCA1/2*	49	OvCa	*BRCA1*	11	c.2005dup	Met669Asnfs*4
AN153	*BRCA1/2*	49	OvCa	*BRCA1*	11	c.2411_2412del	Gln804Leufs*5
AN154	*BRCA1/2*	43	BOC	*BRCA1*	11	c.2486_2487del	Phe829*

AN155	*BRCA1/2*	57	OvCa	*BRCA1*	11	c.2486_2487del	Phe829*
AN156	*BRCA1/2*	48	OvCa	*BRCA1*	11	c.2487del	Phe829Leufs*17
AN157	*BRCA1/2*	61	OvCa	*BRCA1*	11	c.2487del	Phe829Leufs*17
AN158	*BRCA1/2*	42	BrCa	*BRCA1*	11	c.2515del	His839Thrfs*7
AN159	*BRCA1/2*	44	BrCa	*BRCA1*	11	c.2568T>G	Tyr856*
AN160	*BRCA1/2*	49	OvCa	*BRCA1*	11	c.2568T>G	Tyr856*
AN161	*BRCA1/2*	33	BrCa	*BRCA1*	11	c.2800C>T	Gln934*
AN162	*BRCA1/2*	46	BrCa	*BRCA1*	11	c.2921T>A	Leu974*
AN163	*BRCA1/2*	48	BrCa	*BRCA1*	11	c.3193dup	Asp1065Glyfs*2
AN164	*BRCA1/2*	36	BrCa	*BRCA1*	11	c.3228_3229del	Gly1077Alafs*8
AN165	*BRCA1/2*	46	OvCa	*BRCA1*	11	c.3309dup	Lys1104*
AN166	*BRCA1/2*	47	BOC	*BRCA1*	11	c.3700_3704del	Val1234Glnfs*8
AN167	*BRCA1/2*	65	OvCa	*BRCA1*	11	c.3756_3759del	Ser1253Argfs*10
AN168	*BRCA1/2*	39	OvCa	*BRCA1*	11	c.3756_3759del	Ser1253Argfs*10
AN169	*BRCA1/2*	59	OvCa	*BRCA1*	11	c.3758_3759del	Ser1253*
AN170	*BRCA1/2*	23	BrCa	*BRCA1*	11	c.3858_3861del	Ser1286Argfs*20
AN171	*BRCA1/2*	35	OvCa	*BRCA1*	11	c.4042G>T	Gly1348*
AN172	*BRCA1/2*	43	BrCa	*BRCA1*	12	c.4097G>A	Gly1366Asp
AN173	*BRCA1/2*	45	BrCa	*BRCA1*	12	c.4107_4110dup	Gly1371Ilefs*4
AN174	*BRCA1/2*	34	BrCa	*BRCA1*	12	c.4117G>T	Glu1373*
AN175	*BRCA1/2*	45	BrCa	*BRCA1*	12	c.4117G>T	Glu1373*

AN176	*BRCA1/2*	50	OvCa	*BRCA1*	12	c.4117G>T	Glu1373*
AN177	*BRCA1/2*	49	BOC	*BRCA1*	12	c.4128_4129del	Ser1377Argfs*3
AN178	*BRCA1/2*	33	BOC	*BRCA1*	13	c.4327C>T	Arg1443*
AN179	*BRCA1/2*	44	BrCa	*BRCA1*	14	c.4392del	Ile1465*
AN180	*BRCA1/2*	42	OvCa	*BRCA1*	14	c.4484G>A	Arg1495Lys
AN181	*BRCA1/2*	46	BrCa	*BRCA1*	15	c.4925_4926del	Ser1642Tyrfs*36
AN182	*BRCA1/2*	48	OvCa	*BRCA1*	16	c.4688dup	Tyr1563*
AN183	*BRCA1/2*	33	BrCa	*BRCA1*	16	c.4964_4982del	Ser1655Tyrfs*16
AN184	*BRCA1/2*	72	OvCa	*BRCA1*	16	c.4964_4982del	Ser1655Tyrfs*16
AN185	*BRCA1/2*	52	OvCa	*BRCA1*	16i	c.4987-1G>A	
AN186	*BRCA1/2*	43	OvCa	*BRCA1*	17	c.5030_5033del	Thr1677Ilefs*2
AN187	*BRCA1/2*	60	OvCa	*BRCA1*	18	c.5095C>T	Arg1699Trp
AN188	*BRCA1/2*	52	BrCa	*BRCA1*	18	c.5116G>C	Gly1706Arg
AN189	*BRCA1/2*	61	BOC	*BRCA1*	18	c.5123C>A	Ala1708Glu
AN190	*BRCA1/2*	42	BrCa	*BRCA1*	18	c.5123C>A	Ala1708Glu
AN191	AsP	61	OvCa	*BRCA1*	20	c.5266dup	Gln1756Profs*74
AN192	AsP	37	BrCa	*BRCA1*	20	c.5266dup	Gln1756Profs*74
AN193	AsP	45	BrCa	*BRCA1*	20	c.5266dup	Gln1756Profs*74
AN194	*BRCA1/2*	41	BOC	*BRCA1*	20	c.5266dup	Gln1756Profs*74
AN195	*BRCA1/2*	48	BOC	*BRCA1*	20	c.5266dup	Gln1756Profs*74
AN196	*BRCA1/2*	43	BrCa	*BRCA1*	20	c.5266dup	Gln1756Profs*74

AN197	*BRCA1/2*	44	OvCa	*BRCA1*	20	c.5266dup	Gln1756Profs*74
AN198	*BRCA1/2*	45	BrCa	*BRCA1*	20	c.5266dup	Gln1756Profs*74
AN199	*BRCA1/2*	71	OvCa	*BRCA1*	20	c.5266dup	Gln1756Profs*74
AN200	*BRCA1/2*	43	BrCa	*BRCA1*	21	c.5297T>G	Ile1766Ser
AN201	*BRCA1/2*	37	BrCa	*BRCA1*	23	c.5431C>T	Gln1811*
AN202	*BRCA1/2*	48	OvCa	*BRCA1*	23	c.5434C>G	Pro1812Ala
AN203	*BRCA1/2*	44	OvCa	*BRCA1*	23	c.5444G>A	Trp1815*
AN204	MLPA	46	BrCa	*BRCA1*	23i_24_	c.(5467+1_5468-1)_(*1383_?)del	
AN205	*BRCA1/2*	59	OvCa	*BRCA1*	24	c.5509T>C	Trp1837Arg
AN206	*BRCA1/2*	44	OvCa	BRCA1	1-24i	c.(?_-232)_(*1383_?)del	
AN207	*BRCA1/2*	46	BrCa	*BRCA1*	24	c.5522G>A	Ser1841Asn
AN208	*BRCA1/2*	43	BrCa	*BRCA2*	3	c.145G>T	Glu49*
AN209	*BRCA1/2*	36	BrCa	*BRCA2*	7	c.517G>T	Gly173Cys
AN210	*BRCA1/2*	59	BrCa	*BRCA2*	7	c.517G>T	Gly173Cys
AN211	*BRCA1/2*	61	OvCa	*BRCA2*	7	c.517G>T	Gly173Cys
AN212	*BRCA1/2*	55	OvCa	*BRCA2*	10	c.1337T>A	Leu446*
AN213	*BRCA1/2*	67	OvCa	*BRCA2*	10	c.1597del	Thr533Leufs*25
AN214	*BRCA1/2*	66	OvCa	*BRCA2*	10	c.1597del	Thr533Leufs*25
AN215	*BRCA1/2*	37	BrCa	*BRCA2*	10	c.1760del	Thr587Lysfs*27
AN216	*BRCA1/2*	40	BrCa	*BRCA2*	10	c.1814dup	Pro606Thrfs*10
AN217	*BRCA1/2*	59	OvCa	*BRCA2*	10i	c.1909+1G>A	

AN218	*BRCA1/2*	63	OvCa	*BRCA2*	10i	c.1909+1G>A	
AN219	*BRCA1/2*	50	OvCa	*BRCA2*	10i	c.1909+1G>A	
AN220	*BRCA1/2*	60	OvCa	*BRCA2*	10i	c.1909+1G>C	
AN221	*BRCA1/2*	40	BOC	*BRCA2*	11	c.2175del	Val726Phefs*4
AN222	*BRCA1/2*	33	BrCa	*BRCA2*	11	c.2094del	Gln699Serfs*31
AN223	*BRCA1/2*	54	OvCa	*BRCA2*	11	c.2094del	Gln699Serfs*31
AN224	*BRCA1/2*	55	OvCa	*BRCA2*	11	c.2133C>A	Cys711*
AN225	*BRCA1/2*	35	BrCa	*BRCA2*	11	c.2376C>G	Tyr792*
AN226	*BRCA1/2*	63	OvCa	*BRCA2*	11	c.2657delA	Asn886Metfs*9
AN227	*BRCA1/2*	36	BrCa	*BRCA2*	11	c.2808_2811del	Ala938Profs*21
AN228	*BRCA1/2*	38	BrCa	*BRCA2*	11	c.2808_2811del	Ala938Profs*21
AN229	*BRCA1/2*	37	Melanoma	*BRCA2*	11	c.2808_2811del	Ala938Profs*21
AN230	*BRCA1/2*	54	OvCa	*BRCA2*	11	c.2808_2811del	Ala938Profs*21
AN231	*BRCA1/2*	34	BrCa	*BRCA2*	11	c.2836del	Asp946Ilefs*14
AN232	*BRCA1/2*	66	OvCa	*BRCA2*	11	c.2860G>T	Glu954*
AN233	*BRCA1/2*	46	BrCa	*BRCA2*	11	c.3075G>A	Lys1025=
AN234	*BRCA1/2*	48	BrCa	*BRCA2*	11	c.3132T>A	Cys1044*
AN235	*BRCA1/2*	37	BrCa	*BRCA2*	11	c.3403T>C	Tyr1135His
AN236	*BRCA1/2*	53	OvCa	*BRCA2*	11	c.3545_3546del	Phe1182*
AN237	*BRCA1/2*	41	BOC	*BRCA2*	11	c.4131_4132insTGAGGA	Thr1378*
AN238	*BRCA1/2*	58	OvCa	*BRCA2*	11	c.4222C>T	Gln1408*

AN239	*BRCA1/2*	58	OvCa	*BRCA2*	11	c.4284dup	Gln1429Serfs*9
AN240	*BRCA1/2*	56	OvCa	*BRCA2*	11	c.4419del	Asn1473Lysfs*6
AN241	*BRCA1/2*	60	OvCa	*BRCA2*	11	c.4964dup	Tyr1655*
AN242	*BRCA1/2*	59	OvCa	*BRCA2*	11	c.5073dup	Trp1692Metfs*3
AN243	*BRCA1/2*	40	BrCa	*BRCA2*	11	c.5110_5113del	Arg1704*
AN244	*BRCA1/2*	62	OvCa	*BRCA2*	11	c.5130_5133del	Tyr1710*
AN245	*BRCA1/2*	74	OvCa	*BRCA2*	11	c.5146_5149del	Tyr1716Lysfs*8
AN246	*BRCA1/2*	58	OvCa	*BRCA2*	11	c.5146_5149del	Tyr1716Lysfs*8
AN247	*BRCA1/2*	67	BOC	*BRCA2*	11	c.5253C>A	Tyr1751*
AN248	*BRCA1/2*	26	BrCa	*BRCA2*	11	c.5351dup	Asn1784Lysfs*3
AN249	*BRCA1/2*	59	BrCa	*BRCA2*	11	c.5351dup	Asn1784Lysfs*3
AN250	*BRCA1/2*	59	OvCa	*BRCA2*	11	c.5351dup	Asn1784Lysfs*3
AN251	*BRCA1/2*	55	BrCa	*BRCA2*	11	c.5351dup	Asn1784Lysfs*3
AN252	*BRCA1/2*	50	OvCa	*BRCA2*	11	c.5351dup	Asn1784Lysfs*3
AN253	*BRCA1/2*	64	OvCa	*BRCA2*	11	c.5351dup	Asn1784Lysfs3*
AN254	*BRCA1/2*	51	OvCa	*BRCA2*	11	c.5351dup	Asn1784Lysfs*3
AN255	*BRCA1/2*	35	BrCa	*BRCA2*	11	c.5641_5644del	Lys1881Glnfs*27
AN256	*BRCA1/2*	66	OvCa	*BRCA2*	11	c.5723_5724del	Leu1908Argfs*2
AN257	*BRCA1/2*	36	BrCa	*BRCA2*	11	c.5909C>A	Ser1970*
AN258	*BRCA1/2*	46	BrCa	*BRCA2*	11	c.5909C>A	Ser1970*
AN259	AsP	29	BrCa	*BRCA2*	11	c.5946del	Ser1982Argfs*22

AN260	AsP	38	BrCa	*BRCA2*	11	c.5946del	Ser1982Argfs*22
AN261	AsP	43	BrCa	*BRCA2*	11	c.5946del	Ser1982Argfs*22
AN262	AsP	76	BrCa	*BRCA2*	11	c.5946del	Ser1982Argfs*22
AN263	*BRCA1/2*	61	BOC	*BRCA2*	11	c.5946del	Ser1982Argfs*22
AN264	*BRCA1/2*	59	BrCa	*BRCA2*	11	c.5946del	Ser1982Argfs*22
AN265	*BRCA1/2*	36	BrCa	*BRCA2*	11	c.5946del	Ser1982Argfs*22
AN266	*BRCA1/2*	50	BrCa	*BRCA2*	11	c.5946del	Ser1982Argfs*22
AN267	*BRCA1/2*	59	BrCa	*BRCA2*	11	c.5946del	Ser1982Argfs*22
AN268	AsP	44	BrCa	*BRCA2*	11	c.5946del	Ser1982Argfs*22
AN269	AsP	45	BrCa	*BRCA2*	11	c.5946del	Ser1982Argfs*22
AN270	*BRCA1/2*	45	OvCa	*BRCA2*	11	c.5959C>T	Gln1987*
AN271	*BRCA1/2*	49	OvCa	*BRCA2*	11	c.6275_6276del	Leu2092Profs*7
AN272	*BRCA1/2*	49	OvCa	*BRCA2*	11	c.6395T>G	Leu2132*
AN273	*BRCA1/2*	67	BrCa	*BRCA2*	11	c.6405_6409del	Asn2135Lysfs*3
AN274	*BRCA1/2*	25	BrCa	*BRCA2*	11	c.6468_6469del	Gln2157Ilefs*18
AN275	*BRCA1/2*	51	OvCa	*BRCA2*	11	c.6486_6489del	Lys2162Asnfs*5
AN276	*BRCA1/2*	55	OvCa	*BRCA2*	11	c.6596del	Thr2199Ilefs*7
AN277	*BRCA1/2*	37	BOC	*BRCA2*	11	c.6833_6837del	Ile2278Serfs*13
AN278	*BRCA1/2*	33	BrCa	*BRCA2*	13	c.7007G>A	Arg2336His
AN279	*BRCA1/2*	31	BrCa	*BRCA2*	13	c.7007G>A	Arg2336His
AN280	*BRCA1/2*	35	BOC	*BRCA2*	14	c.7308del	Asn2436Lysfs*33

AN281	*BRCA1/2*	48	BrCa	*BRCA2*	15	c.7480C>T	Arg2494*
AN282	*BRCA1/2*	47	BrCa	*BRCA2*	17	c.7940T>C	Leu2647Pro
AN283	*BRCA1/2*	53	BrCa	*BRCA2*	17	c.7940T>C	Leu2647Pro
AN284	*BRCA1/2*	50	OvCa	*BRCA2*	17	c.7976G>A	Arg2659Lys
AN285	*BRCA1/2*	48	BrCa	*BRCA2*	18	c.8009C>T	Ser2670Leu
AN286	*BRCA1/2*	32	BrCa	*BRCA2*	18	c.8168A>G	Asp2723Gly
AN287	*BRCA1/2*	49	BrCa	*BRCA2*	18	c.8168A>G	Asp2723Gly
AN288	*BRCA1/2*	44	BOC	*BRCA2*	18	c.8247_8248del	Lys2750Aspfs*13
AN289	*BRCA1/2*	44	BrCa	*BRCA2*	19	c.8463dup	Ile2822Tyrfs*23
AN290	*BRCA1/2*	49	OvCa	*BRCA2*	19i	c.8488-1G>T	
AN291	*BRCA1/2*	40	OvCa	*BRCA2*	19i	c.8488-1G>T	
AN292	*BRCA1/2*	33	BrCa	*BRCA2*	21i	c.8755-1G>A	
AN293	*BRCA1/2*	45	BOC	*BRCA2*	22	c.8784_8801delinsAGTTAAG	Leu2929Valfs*6
AN294	*BRCA1/2*	56	OvCa	*BRCA2*	23	c.8987T>A	Leu2996*
AN295	*BRCA1/2*	46	BOC	*BRCA2*	23	c.9026_9030del	Tyr3009Serfs*7
AN296	*BRCA1/2*	45	BrCa	*BRCA2*	23	c.9069_9076del	Asn3024Valfs*17
AN297	*BRCA1/2*	39	BOC	*BRCA2*	23i	c.9118-1G>A	
AN298	*BRCA1/2*	42	BrCa	*BRCA2*	25	c.9455_9456del	Glu3152Glyfs*15

BRCA1/2: NGS sequencing for *BRCA1* and *BRCA2* genes.

AsP: Ashkenazi panel including the following pathogenic variants: c.68_69del and c.5266dup and c.5946del in *BRCA2.*

MLPA: MLPA for *BRCA1/2.*

NO: no cancer detected.

BrCa: breast cancer.

OvCa: Ovary cancer.

BOC: Breast and ovary cancer.

Age+SD (number of cases) for both genes in:.

BRCA1: BrCa, 44.0+8.9 (n=39); OvCa: 52.9+10.3 (n=41); BOC, 45.6+7.9 (n=8) and No tumor: 38.1+9.5 (n=12).

BRCA2: BrCa, 43.0+10.6 (n=45); OvCa: 56.9+6.9 (n=36); BOC, 45.5+9.9 (n=10) and No tumor: 50.5+8.3 (n=6).

Table S2. Male individuals with pathogenic variant detected without cancer manifestation, relatives of the probands in the cohort and not included in the 2155 individuals. Total listed = 40; age, years, mean ± SD (range): 42.5 ± 12.9 (19–71).

ID	Age	Gene	Exon / Intron	HGVS c.	HGVS p.
AN300	44	*BRCA1*	2	c.68_69del	p.Glu23Valfs*17
AN301	71	*BRCA1*	2	c.68_69del	p.Glu23Valfs*17
AN302	62	*BRCA1*	2	c.68_69del	p.Glu23Valfs*17
AN303	36	*BRCA1*	5	c.140G>T	Cys47Phe
AN304	36	*BRCA1*	5	c.140G>T	Cys47Phe
AN305	53	*BRCA1*	11	c.1067del	Gln356Argfs*18
AN306	24	*BRCA1*	11	c.1674del	Gly559Valfs*13
AN307	45	*BRCA1*	11	c.3001G>T	Glu1001*
AN308	61	*BRCA1*	11	c.4042G>T	Gly1348*
AN309	50	*BRCA1*	13	c.4201C>T	Gln1401*
AN310	47	*BRCA1*	16	c.4964_4982del	Ser1655Tyrfs*16
AN311	42	*BRCA1*	16i	c.4987-1G>A	
AN312	35	*BRCA1*	18	c.5123C>A	Ala1708Glu
AN313	47	*BRCA1*	23	c.5434C>G	Pro1812Ala
AN314	66	*BRCA1*	23	c.5434C>G	Pro1812Ala
AN315	38	*BRCA2*	2	c.51_52del	Arg18Leufs*12
AN316	37	*BRCA2*	2	c.51_52del	Arg18Leufs*12
AN317	64	*BRCA2*	2	c.66A>C	Ala22=
AN318	21	*BRCA2*	3	c.124del	Tyr42Ilefs*38
AN319	59	*BRCA2*	10	c.1670T>G	Leu557*
AN320	45	*BRCA2*	11	c.2808_2811del	Ala938Profs*21
AN321	36	*BRCA2*	11	c.2808_2811del	Ala938Profs*21
AN322	42	*BRCA2*	11	c.2808_2811del	Ala938Profs*21
AN323	34	*BRCA2*	11	c.2808_2811del	Ala938Profs*21
AN324	45	*BRCA2*	11	c.2808_2811del	Ala938Profs*21
AN325	60	*BRCA2*	11	c.3187C>T	Gln1063*
AN326	21	*BRCA2*	11	c.4131_4132insTGAGGA	Thr1378*
AN327	40	*BRCA2*	11	c.5351dup	Asn1784Lysfs*3
AN328	38	*BRCA2*	11	c.5351dup	Asn1784Lysfs*3
AN329	38	*BRCA2*	11	c.5681dup	Tyr1894*
AN330	38	*BRCA2*	11	c.5681dup	Tyr1894*

AN331	38	*BRCA2*	11	c.5796_5797del	His1932Glnfs*12
AN332	43	*BRCA2*	11	c.5946del	Ser1982Argfs*22
AN333	23	*BRCA2*	11	c.5946del	Ser1982Argfs*22
AN334	19	*BRCA2*	11	c.5946del	Ser1982Argfs*22
AN335	26	*BRCA2*	13	c.7007G>A	Arg2336His
AN336	41	*BRCA2*	14	c.7308del	Asn2436Lysfs*33
AN337	56	*BRCA2*	17	c.7857G>A	Trp2619*
AN338	56	*BRCA2*	17	c.7857G>A	Trp2619*
AN339	33	*BRCA2*	25	c.9498del	Glu3167Argfs*50
